# Gonadal and sexual function in men living with HIV: insights from a single-centre study

**DOI:** 10.1007/s40618-025-02683-5

**Published:** 2025-09-22

**Authors:** Ilaria Bonaventura, Valeria Hasenmajer, Nicolò F. D’Addario, Carlotta Pozza, Giancarlo Ceccarelli, Gabriella d’Ettorre, Claudio M. Mastroianni, Emmanuele A. Jannini, Daniele Gianfrilli

**Affiliations:** 1 Department of Experimental Medicine, Sapienza University of Rome, Viale del Policlinico 155, Rome, 00161 Italy; 2 Department of Public Health and Infectious Diseases, Sapienza University of Rome, Rome, Italy; 3 Department of Systems Medicine, Tor Vergata University of Rome, Rome, Italy

**Keywords:** Erectile dysfunction, HIV, Hypogonadism, Sex steroids, Sexual dysfunction, Testosterone.

## Abstract

**Purpose:**

The study aimed to estimate the prevalence of hypogonadism and erectile dysfunction (ED) in male living with the human immunodeficiency virus (HIV), MLWH, and to explore associations between HIV-related variables and gonadal/sexual function.

**Methods:**

From 2019 to 2024, gonadal and sexual function were evaluated in consecutively enrolled MLWH through hormonal assessments and IIEF-15 questionnaire. Anthropometrics and HIV-related parameters, including type of Highly Active Anti-Retroviral Therapy, HAART, were also evaluated.

**Results:**

Among 60 MLWH, 70.0% presented with ED. Hypogonadism was observed in 18.3%, primarily hypogonadotropic (72.7%). Although both eu- and hypogonadal MLWH presented pathological IIEF-15 scores, no differences in the five domains of IIEF-15 were found. Hypogonadal MLWH had significantly higher BMI (*p* = 0.046) and greater smoking prevalence (*p* = 0.002), and lower 17β-estradiol levels (*p* = 0.017). In the whole cohort, total testosterone was negatively correlated to BMI (*r*=-0.595, *p* = 0.001) and waist circumference (*r*=-0.656, *p* = 0.011), and positively to 17β-estradiol (*r* = 0.457, *p* = 0.006) and SHBG (*r* = 0.325, *p* = 0.033). Calculated free testosterone also negatively correlated with BMI (*r*=-0.519, *p* = 0.023) and WC (*r*=-0.719, *p* = 0.019). Considering HAART, ED was more prevalent among those using Integrase Strand Transfer Inhibitor (*p* = 0.017). Conversely, MLWH treated with Proteinase Inhibitors showed higher total testosterone, SHBG and 17β-estradiol levels (respectively, *p* = 0.018, *p* = 0.015 and *p* = 0.020), despite no differences in calculated free testosterone or prevalence of ED.

**Conclusion:**

ED is highly prevalent multifactorial disorder in MLWH. Decreased serum testosterone levels, which are also related to increased visceral fat accumulation, are not the only driver of its onset. HIV-related factors, such as HAART, also appear to have an impact on gonadal and sexual function. A multidisciplinary approach, integrating infectious disease and sexual medicine expertise, is essential for optimal care.

**Supplementary Information:**

The online version contains supplementary material available at 10.1007/s40618-025-02683-5.

## Introduction

Innovations in lifesaving Highly Active Anti-Retroviral Therapy (HAART) and access to effective prevention, diagnosis, and care have converted human immunodeficiency virus (HIV) infection into a manageable chronic disease, enabling people living with HIV (PLWH) to experience long and healthy lives [1]. Although the life expectancy of PLWH is now comparable to that of the general population, PLWH remain vulnerable to the development of hormonal and sexual complications [2–4]. These complications arise not only because of increased life expectancy, but also as a result of viral factors, immune-related conditions, and, in particular, the HAART used to treat HIV infection. Therefore, as PLWH live longer thanks to HAART, it is important to be aware of these conditions that may affect the quality of life and overall life expectancy [2]. Hypogonadism commonly affects men living with HIV (MLWH), although the prevalence remains poorly defined and widely ranging from 13 to 40%, depending on the population studied and the diagnostic criteria used [5–8]. Variability between studies is mostly due to differences in age distribution, different cut-offs used to define low serum total testosterone (TT), different laboratory methods used to measure TT, and whether TT or calculated free testosterone (cFT) is used - the latter being a more valid indicator in MLWH due to frequently elevated sex hormone-binding globulin (SHBG) levels [6, 8, 9]. According to current guidelines, detailed and accurate evaluation of pituitary-gonadal axis function in MLWH should therefore require measurement of SHBG along with serum TT and gonadotropins [10–12]. Nonetheless, the diagnosis of hypogonadism in MLWH remains challenging, as signs and symptoms are non-specific, mild to moderate in severity, and often overlap with those of other conditions in MLWH [8].

A healthy and satisfying sexual life is considered crucial for overall well-being and a satisfactory quality of life [13]. Despite recognition of its importance to PLWH, sexual problems are often neglected in HIV management. In men, sexual dysfunction, particularly erectile dysfunction (ED), is also more prevalent in HIV-infected than HIV-uninfected men [14, 15]. Sexual dysfunction is multifactorial, and in addition to traditional risk factors such as age, and lifestyle-dependent non-communicable chronic diseases (NCDs), several HIV-related factors such as HAART and emotional and psychological factors (anxiety and depression) play a role in its pathogenesis [16, 17]. Despite its clinical relevance, few studies have comprehensively investigated sexual dysfunction and gonadal status through complete hormonal profile in MLWH. This study aims to bridge this knowledge gap by describing the prevalence of hypogonadism and sexual dysfunction in a cohort of MLWH and to explore the influence of HIV-related factors on gonadal and sexual function.

## Materials and methods

### Patient selection

This was a cross-sectional observational study of prospectively collected data from January 2019 to December 2024, enrolling patients with HIV infections referred to the integrated outpatient clinic of Endocrinology and Andrology of the Department of Experimental Medicine, Policlinico Umberto I, Sapienza University of Rome. Only MLWH were included in the study. Women living with HIV were excluded, along with MLWH treated with androgens, gonadotropins, or drugs with potential detrimental role on gonadal function. Moreover, MLWH with chronic renal failure, chronic hepatic disease, active malignancy, and acquired immunodeficiency syndrome (AIDS) were also excluded from the study. Inclusion criteria were therefore serologically documented HIV infection in men in stable condition under HAART with availability of HIV infection parameters such as time since HIV diagnosis, duration of HAART and CD4^+^ T helper cell count. Duration of exposure to the current HAART regimen (in months) was collected for all participants at the time of data collection. Anthropometric measurements included body mass index (BMI), calculated by dividing weight (in kg) by squared height (in m^2^) and waist circumference (WC). This study was approved by the Local Ethics Committee of Sapienza University in Rome (reference number 5681, Prot.15/2020) and was conducted in accordance with the Declaration of Helsinki (1964) and its subsequent amendments. All enrolled patients provided their written informed consent to participate in the study.

### Gonadal function evaluation

All biochemical tests were performed locally at the study centre. Gonadal function was studied by measurement of the following hormones: follicle stimulating hormone (FSH), luteinizing hormone (LH), TT, 17β-estradiol (E2) and SHBG. Blood samples were obtained via antecubital venous puncture in the early morning (7:30 − 9:30 am) after overnight fasting. Samples were centrifuged, and the serum was immediately frozen at − 20 °C. FSH, LH, TT, E2, and SHBG levels were measured in duplicate using the chemiluminescent microparticle immunoassay (Architect System, Abbott Laboratories, IL, USA, catalog no. 7K75-25, RRID: AB_2813910; catalog no. 2P40-25, RRID: AB_2813909; catalog no. 2P13, RRID: AB_2895254; catalog no. 7K72-25, RRID: AB_2813911; and catalog no. 8K26, RRID: AB_2895255, respectively) with limits of detection of 0.07 mIU/mL, 0.05 mIU/mL, 0.1 nmol/L, 10 pg/mL, and 0.28 nmol/L, respectively. The intra- and interassay coefficients of variation were as follows: 3.8% and 5.5% at 4.1 mIU/mL (LH), 3.6% and 5.4% at 3.2 mIU/mL (FSH), 2.1% and 3.6% at 10.08 nmol/L (TT), 5% and 7% at 190 and 600 pg/mL (E2), and 5.65% and 9.54% at 8.8 nmol/L (SHBG), respectively. We used Vermeulen’s formula to calculate the cFT from TT and SHBG levels [18]. In accordance with the EAA guidelines, diagnosis of biochemical hypogonadism was made with serum TT ≤ 12 nmol/L and/or cFT < 0.22 nmol/L [12].

### Sexual function assessment

Sexual function was assessed through the International Index of Erectile Function-15 (IIEF-15), that is the most used validated questionnaire for ED in clinical practice [19]. It is a self-reported questionnaire that investigates the five domains of male sexual function in the last month: erectile function (EF), ejaculatory and orgasmic function, sexual desire, intercourse satisfaction, and overall satisfaction with sex life. The score is structured on a 5-point scale (from the worst, 1, to the best score of 5) and a cumulative score of EF domain below or equal to 25 is used to diagnose ED [19, 20]. According to the score of the EF domain, the EF was classified into the following four diagnostic categories: no ED (EF score = 26–30), mild ED (EF score = 17–25), moderate ED (EF score = 11–16), and severe ED (EF = 6–10). Sexual desire, orgasmic function, intercourse satisfaction, and overall satisfaction were defined as impaired with a score below 9 at the specific IIEF-15 domains [19 , 20].

### Statistical analysis

Distribution of continuous variables was assessed with the Shapiro–Wilk test; linearity was established by visual inspection of a scatterplot. Categorical variables are expressed as percentage and frequency; continuous variables are reported as mean and standard deviation (SD) or median and interquartile range (IQR − 25th −75th percentile) as appropriate per distribution of data. For group comparisons unpaired Student’s T-test, Mann–Whitney, χ2 or Fisher’s exact test were used as appropriate. For all comparisons, *p* < 0.05 was considered statistically significant. Statistical analyses were performed using SPSS, version 29 (IBM, Chicago, IL) and GraphPad Prism 10.0 software package (GraphPad Software, La Jolla, CA).

## Results

### Characteristics of the whole cohort

Between January 2019 to December 2024, out of 93 MLWH referred to our department, 60 met the study inclusion criteria. The mean age of the participants was 56 ± 9 years, with a mean BMI and WC respectively of 28.4 ± 5.6 kg/m² and 106 ± 16 cm. The mean duration of HIV infection was 19 ± 10 years, and all subjects were in stable clinical conditions under HAART, with viral suppression and adequate median CD4^+^ T helper cell count of 686 [484–917] cells/µL at time of investigation. Regarding the type of antiretroviral used, 78.3% used Nucleoside Reverse Transcriptase Inhibitor (NRTI), 20% Non-Nucleoside Reverse Transcriptase Inhibitor (NNRTI), 11.7% Protease Inhibitor (PI) and 71.7% Integrase Strand Transfer Inhibitor (INSTI). The median duration of exposure to the current HAART regimen was 18.5 months [7.5–40.0]. A summary of characteristics of the whole cohort is shown in Table 1.

**Table 1:** Clinical characteristics of the whole cohort

	All (*n* = 60)
Age (years)	56 ± 9
Duration of HIV infection (years)	19 ± 10
Active smoking (%)	23 (38.3)
BMI (kg/m²)	28.4 ± 5.6
WC (cm)	106 ± 16
CD4^+^ (cells/µL)	686 [484–917]
Exposure to current HAART (months)	18.5 [7.5–40.0]
NRTI (%)	47 (78.3)
NNRTI (%)	12 (20)
PI (%)	7 (11.7)
INSTI (%)	43 (71.7)
TT (nmol/L)	17.3 ± 7.1
cFT(nmol/L)	0.33 [0.25–0.39]
SHBG (nmol/L)	47 [35.2–55.1]
LH (mUI/mL)	5.5 [3.4–7.2]
E2 (pg/mL)	26.0 ± 8.4
Hypogonadism (%)	11 (18.3)
IIEF-15 Erectile Function (n.v. >25)	21 [11–26]
IIEF-15 Orgasmic Function (n.v. >9)	9 [5–10]
IIEF-15 Sexual Desire (n.v. >9)	8 [6–10]
IIEF-15 Intercourse Satisfaction (n.v. >9)	6 [3–10]
IIEF-15 Overall Satisfaction (n.v. >9)	9 [4–10]
ED (%)	42 (70.0)
Impaired orgasmic function (%)	39 (65.0)
Impaired sexual desire (%)	41 (68.3)
Impaired intercourse satisfaction (%)	43 (71.7)
Impaired overall satisfaction (%)	41 (68.3)

Continuous data are expressed as mean ± SD or median [IQR], as appropriate. Categorical variables are expressed as frequency (%)

Abbreviations: Human immunodeficiency virus (HIV); BMI, body mass index; WC, waist circumference; CD4^+^, CD4^+^ T helper cell count; NRTI, Nucleoside Reverse Transcriptase Inhibitor; NNRTI, Non-Nucleoside Reverse Transcriptase Inhibitor; PI, Protease Inhibitor; INSTI, Integrase Strand Transfer Inhibitor; TT, total testosterone; cFT, calculated free testosterone; SHBG, sex hormone binding globulin; LH, luteinizing hormone; E2, estradiol; ED, erectile disfunction; IIEF-15, International Index of Erectile Function-15; n.v., normal value

### Gonadal and sexual function of the whole cohort

**Fig. 1:**
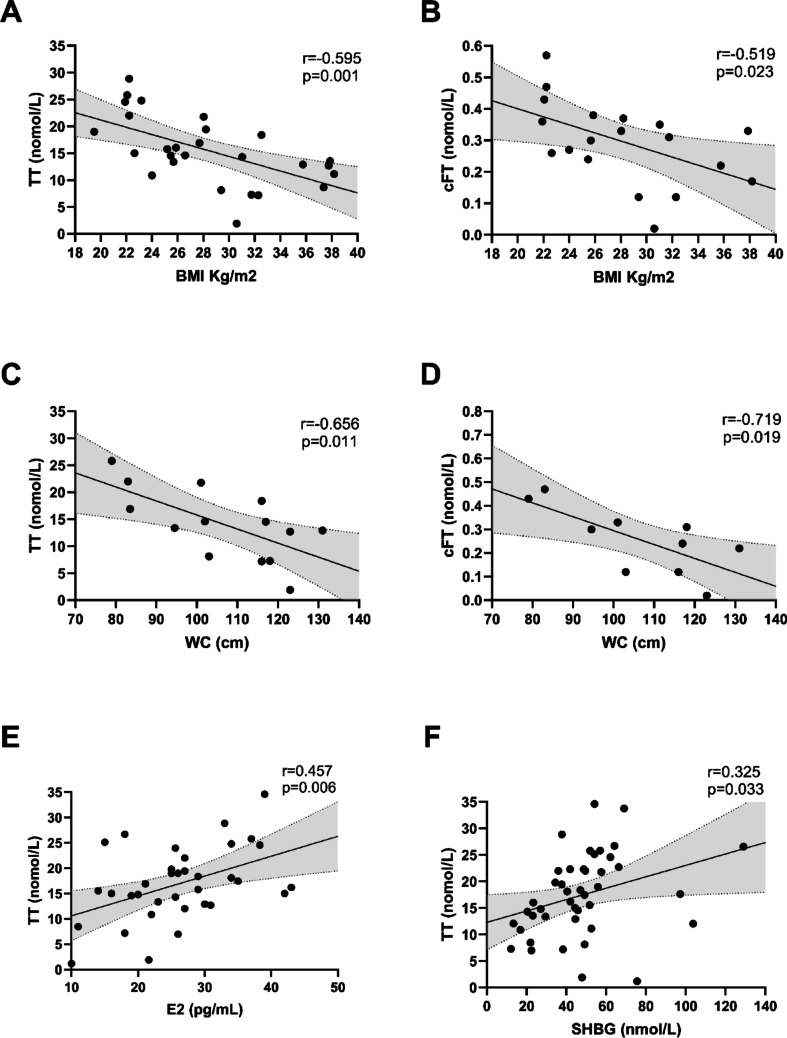
Univariate analysis in the entire cohort showing the relationship between TT and BMI **(A)**, cFT and BMI **(B)**, TT and WC **(C)**, cFT and WC **(D)**, TT and E2 **(E)**, and TT and SHBG **(F)**. The grey area between the dashed lines represents the 95% confidence intervals of the regression lines. Abbreviations: BMI, body mass index; cFT, calculated free testosterone; E2, estradiol; SHBG, sex hormone binding globulin; TT, total testosterone; WC, waist circumference

**Fig. 2:**
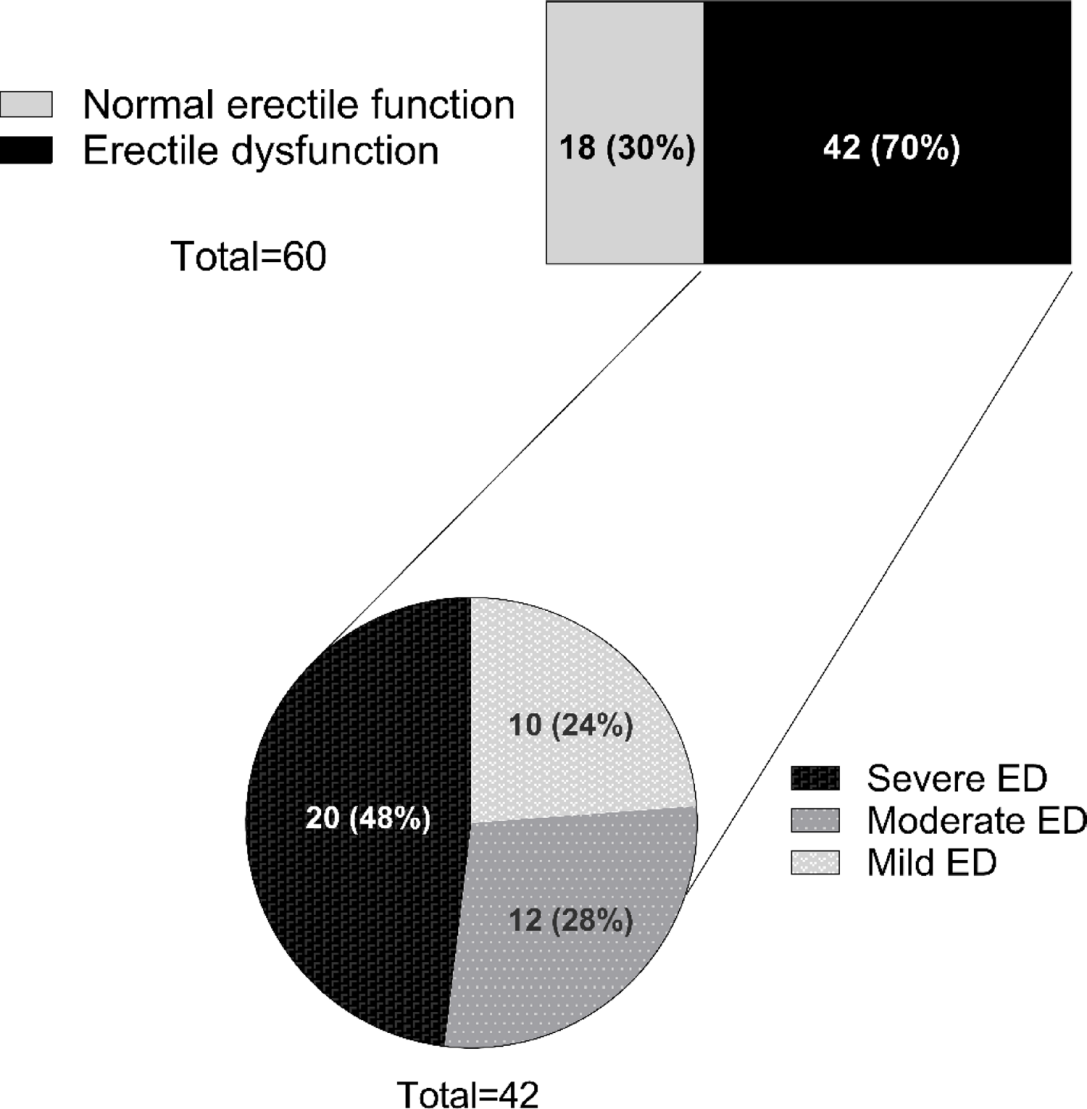
Prevalence and distribution of severity of erectile dysfunction according to IIEF-15 domain. Abbreviations: IIEF, International Index of Erectile Function; ED, erectile dysfunction

According with the guidelines [12], diagnosis of biochemical hypogonadism was present in 18.3% of the entire cohort, of which 72.7% had secondary hypogonadism (Table 1). Among the 11 patients classified as hypogonadal based on cFT, 3 (27.3%) had TT levels above the conventional diagnostic threshold and would have been missed if SHBG had not been considered. A univariate analysis across the whole cohort revealed that TT and cFT were negatively correlated to BMI (*r *= −0.595, *p* = 0.001 and *r *= −0.519, *p* = 0.023, respectively) and WC (*r *= −0.656, *p* = 0.011 and *r *= −0.719, *p* = 0.019, respectively). Moreover, TT positively correlated to E2 (*r* = 0.457, *p* = 0.006) and SHBG (*r* = 0.325, *p* = 0.033), as shown in Fig. 1. According to IIEF-15 scores, 70.0% presented ED: 48.0% had severe ED, 28.0% had moderate ED, and 24.0% had mild ED (Table 1; Fig. 2). Moreover, when considering domains other than erectile function, 65.0% of participants had impaired orgasmic function, 68.3% had impaired sexual desire, 71.7% had impaired intercourse satisfaction and 68.3% had impaired overall satisfaction. No patients with hypogonadism were receiving testosterone replacement therapy, and no patients were taking phosphodiesterase-5 inhibitors at the time of the investigation.

### Sub-group analysis based on the presence or absence of hypogonadism and erectile dysfunction

Eleven patients were classified as hypogonadal (18.3%), and forty-nine were in a normal eugonadal state (81.7%). Both groups were comparable in terms of age, duration of HIV infection and CD4^+^ T helper cell count. However, patients with hypogonadism exhibited higher BMI (31.9 ± 4.8 kg/m² vs. 27.1 ± 5.4 kg/m², *p* = 0.046), a greater prevalence of smoking habits (81.8% vs. 28.6%, *p* = 0.002), and lower E2 levels (19.4 ± 6.7 pg/mL vs. 27.6 ± 8.1 pg/mL, *p* = 0.017) compared to eugonadal patients, as shown in Table 2. Interestingly, although pathological IIEF-15 scores were observed in both groups, no differences were found in the prevalence of ED and in the five domains of the IIEF-15 between MLWH with hypogonadism and those with eugonadism (Table 2). According to IIEF-15 scores, no differences in gonadal status were observed between patients with ED and those with normal erectile function (Table S1).

**Table 2:** Clinical characteristics stratified for presence or absence of hypogonadism

	Eugonadism (*n* = 49)	Hypogonadism (*n* = 11)	*p*-value
Age (years)	56 ± 9	58 ± 9	0.471
Duration of HIV infection (years)	19 ± 9	19 ± 11	0.864
Active smoking (%)	14 (28.6)	9 (81.8)	**0.002**
BMI (kg/m**²**)	27.1 ± 5.4	31.9 ± 4.8	**0.046**
WC (cm)	103 ± 18	115 ± 9	0.121
CD4^+^ (cells/µL)	690 [486–917]	484 [354–1098]	0.657
TT (nmol/L)	19.8 ± 5.5	7.6 ± 3.4	**< 0.001**
cFT(nmol/L)	0.35 [0.30–0.42]	0.14 [0.10–0.27]	**< 0.001**
SHBG (nmol/L)	47.0 [37.7–55.9]	43.1 [22.0-52.5]	0.419
LH (mUI/mL)	5.2 [3.4–7.1]	6.6 [3.6–11.6]	0.282
E2 (pg/mL)	27.6 ± 8.1	19.4 ± 6.7	**0.017**
IIEF-15 Erectile Function (n.v. >25)	24 [8–26]	18 [10–26]	0.694
IIEF-15 Orgasmic Function (n.v. >9)	9 [4–10]	8 [2–10]	0.848
IIEF-15 Sexual Desire (n.v. >9)	9 [5–10]	8 [6–10]	0.593
IIEF-15 Intercourse Satisfaction (n.v. >9)	6 [3–10]	7 [1–10]	0.988
IIEF-15 Overall Satisfaction (n.v. >9)	9 [6–10]	8 [3–10]	0.480
ED (%)	34 (69.4)	8 (72.7)	0.846
Impaired orgasmic function (%)	31 (63.3)	8 (72.7)	0.732
Impaired sexual desire (%)	33 (67.3)	8 (72.7)	0.616
Impaired intercourse satisfaction (%)	35 (71.4)	8 (72.7)	0.619
Impaired overall satisfaction (%)	33 (67.3)	8 (72.7)	0.732

Continuous data are expressed as mean ± SD or median [IQR], as appropriate. Categorical variables are expressed as frequency (%). Boldfaced p values are significant

Abbreviations: Human immunodeficiency virus (HIV); BMI, body mass index; WC, waist circumference; CD4^+^, CD4^+^ T helper cell count; TT, total testosterone; cFT, calculated free testosterone; SHBG, sex hormone binding globulin; LH, luteinizing hormone; E2, estradiol; ED, erectile disfunction; IIEF-15, International Index of Erectile Function-15; n.v., normal value

### Sub-group analysis based on type of HAART used

**Fig. 3:**
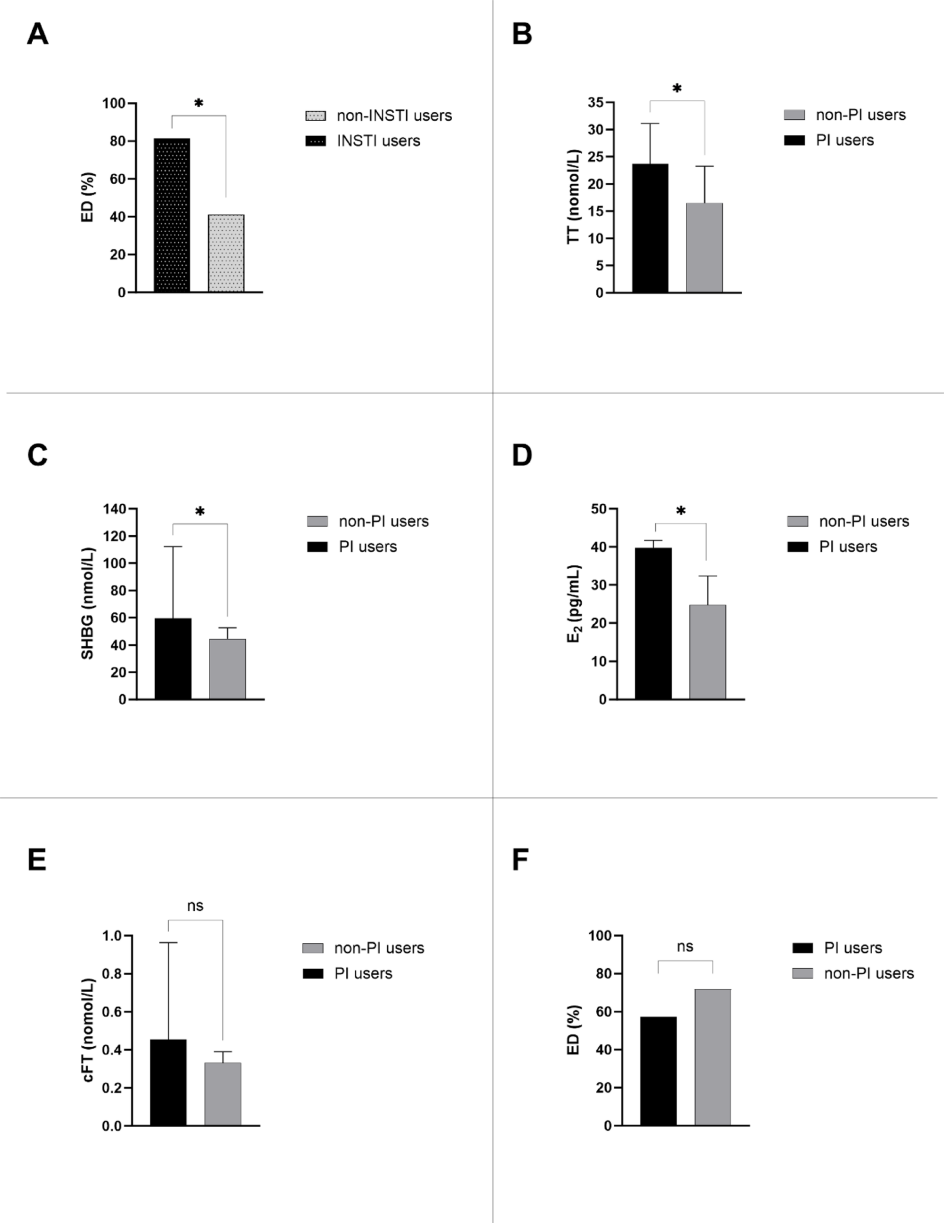
Comparison of erectile dysfunction prevalence and hormonal parameters between INSTI and PI users and their non-user counterparts. (**A**) ED prevalence in INSTI users compared to non-INSTI users. (**B**) Total testosterone levels in PI users compared to non-PI users. (**C**) Sex hormone-binding globulin in PI users. (**D**) Estradiol levels in PI users compared to non-PI users. (**E**) Calculated free testosterone in PI users compared to non-PI users. (**F**) ED prevalence in PI users compared to non-PI users. Continuous data are expressed as mean ± SD or median [IQR], as appropriate. Categorical variables are expressed as frequency (%). Significant P-values are highlighted with an asterisk; ns = not significant

Prevalence of ED was higher in MLWH using INSTI compared to non-INSTI users (81.4% vs. 41.2%, *p* = 0.017), as shown in Fig. 3A and Table S2. Moreover, higher levels of TT, SHBG, and E2 were found in MLWH using PI compared to non-PI users (respectively, 23.7 ± 7.4 nmol/L vs. 16.5 ± 6.8 nmol/L, *p* = 0.018; 59.6 [55.6–95.5] nmol/L vs. 45.1 [29.5–52.5] nmol/L, *p* = 0.015; 39.8 ± 2.0 pg/mL vs. 24.5 ± 7.5 pg/mL, *p* = 0.020), as shown in Fig. 3B-D and Table S3. However, no differences in cFT or prevalence of ED were found between these groups (respectively, 0.45 [0.24–0.96] nmol/L vs. 0.32 [0.24–0.39] nmol/L, *p* = 0.268, and 57.1% vs. 71.7%, *p* = 0.207), as shown in Fig. 3E-F and Table S3.

## Discussion

This study investigates gonadal and sexual function in a homogeneous cohort of MLWH, describing the prevalence of hypogonadism and ED and exploring the influence of HIV-related variables on hormonal and sexual aspects. Despite the high prevalence of ED in this cohort, the decrease in serum testosterone levels, which is also correlated with increased visceral fat accumulation, is not the sole cause of its onset. HIV-related factors, such as the type of HAART used, also appear to impact on gonadal and sexual function. In our cohort, hypogonadism was observed in approximately 20% of the patients, mostly due to hypogonadotropic hypogonadism. Our findings are consistent with previous studies that also included SHBG levels, demonstrating that the prevalence of hypogonadism is underestimated when only TT levels are considered, compared to cFT accounting for SHBG, which can be elevated in these patients [6, 21]. This prevalence is higher than that reported in MLWH younger than 50 years, underlying that age is an important variable influencing the onset of hypogonadism in this cohort, as in the general population [22]. However, compared to the general population, our study confirmed that the prevalence of hypogonadism is higher in MLWH [23]. Among the analysed variables, visceral adipose tissue, in terms of BMI and WC, was inversely related to testosterone level in MLWH. These results suggest that adipose tissue may be involved in the suppression of the hypothalamic-pituitary axis, leading to a decrease in serum LH in MLWH, similarly to what has been reported in non-infected obese men and may contribute to the onset of hypogonadotropic hypogonadism [11, 24–26]. Interestingly, in our cohort, TT was positively correlated with E2, highlighting that lower testosterone levels are associated with lower E2 concentrations. This is consistent with the role of circulating androgens as substrates for aromatase, the key enzyme responsible for converting testosterone into estrogens. Therefore, low E2 levels may reflect reduced aromatization due to decreased circulating testosterone levels, as reported in the literature [27–29]. In our cohort, ED was highly prevalent, reaching 70%, and in nearly half of the cases, it was of severe grade, in line with previous studies on ED in MLWH [14, 16, 30–34]. Interestingly, ED prevalence in our cohort was more than 4-time higher than the age-matched Italian general population [35]. Despite the high prevalence of ED observed in our cohort, no participant reported the use of PDE5i. This may reflect an underdiagnosed or underreported condition, possibly related to low treatment-seeking behavior, limited awareness of available therapies, stigma, or the perception that ED is an unavoidable or secondary issue in the context of chronic HIV infection. This finding suggests a potentially unmet clinical need that should be further explored. However, the most relevant finding of our study was that prevalence of ED did not differ between eugonadal and hypogonadal MLWH, with both groups exhibiting pathological IIEF-15 scores. Similarly, when the cohort was stratified according to the presence or absence of ED (as determined by IIEF-15 scores), the prevalence of hypogonadism remained comparable between the two groups.

This confirms that gonadal status has a limited impact on ED, suggesting that other androgen-independent factors may contribute to the onset of ED in this population [16, 24, 34, 36, 37]. Among these factors, endothelial dysfunction might play a key role. The vascular component of erectile function is well established, and endothelial health is critical for proper penile blood flow [13, 38–41]. In MLWH, chronic inflammation, immune activation, and some antiretroviral therapies may contribute to endothelial dysfunction, potentially linking cardiovascular impairment and ED in a way that is independent of gonadal status [42, 43]. In lines with this consideration, young MLWH showed a significantly higher prevalence of pathological intima-media thickness of the right and left cavernous arteries compared to age-matched HIV-negative controls, aligning with vascular profiles of older individuals [44]. This suggests a premature onset of vascular aging in MLWH, possibly contributing to ED irrespective of gonadal function. In this context, penile doppler ultrasound may represent a valuable diagnostic tool to better characterize the vascular component of ED in MLWH [44]. In addition to differentiating vascular from non-vascular ED, it may also help identify individuals at increased cardiovascular risk, thereby providing an opportunity for early intervention [44, 45]. Future studies should consider integrating penile doppler ultrasound into the diagnostic workup of ED in this population. Indeed, unlike in HIV-uninfected individuals, where serum testosterone levels are correlated with sexual function, this relationship is not as clear in MLWH [10], indicating that other HIV-related factors may influence the onset of ED. Although the IIEF-15 questionnaire allowed us to quantify the severity of ED, it does not provide information on its underlying causes. The Structured Interview on Erectile Dysfunction (SIEDY), which offers a multidimensional assessment of ED by evaluating organic, relational, and psychogenic components, could help better characterize the complex pathophysiology of ED in this population, where multiple factors may coexist and interact [46, 47]. A recent application of the SIEDY in MLWH showed that 45.0% of individuals presented a psychological component contributing to ED (scale 3), and notably, 34.8% of them did not yet report ED (according to IIEF-15) [16]. Patients with ED had significantly higher SIEDY scale 3 score compared to those without ED, suggesting a predominant role of the psychological factors beyond gonadal status in the development of ED in this population [16].

Notably, sexual dysfunctions have become increasingly common following the introduction of HAART. However, the exact mechanisms involved, as well as the specific drugs that exert the greatest influence, remain unclear [48–50]. In our cohort, patients using INSTIs exhibited a higher prevalence of ED compared to non-INSTI-users. This was the only observed difference between the two groups, as they had comparable age, smoking habits, BMI, and gonadal function, all factors known to influence ED. To date, evidence regarding a possible association between INSTIs use and sexual dysfunction is lacking. Nevertheless, it is plausible that the metabolic effects of these drugs may contribute to this observation, despite findings in this area being inconsistent [51–53]. In fact, INSTIs have been associated with significant weight gain and metabolic syndrome, conditions known to impair endothelial nitric oxide production and to promote oxidative stress and inflammation, all of which can compromise penile vascular function [54, 55]. Furthermore, signs of premature vascular aging in MLWH, such as increased intima-media thickness of cavernous arteries, may be exacerbated by such metabolic dysregulation [13]. Consequently, INSTI-related metabolic alterations could impair endothelial health in the penile vasculature, contributing to ED through androgen-independent mechanisms. Future longitudinal studies, ideally incorporating biomarkers of endothelial function and metabolic profiling, are warranted to better elucidate these pathways. On the other hand, PI users did not have a higher frequency of sexual dysfunction or worse gonadal function compared to non-users. Among antiretroviral drugs, PIs have been the most extensively studied in relation to sexual dysfunctions. Several studies have found a correlation between ED and PI utilization, although in most cases, ED was investigated without using standardized questionnaires [48, 49, 56–58]. Conversely, other studies using the IIEF questionnaire did not find an association between ED and the use of PIs [59–61]. In summary, although sexual dysfunction has been widely reported in association with PI regimens, the studies were mainly not controlled studies with heterogeneous methods for assessing sexual disfunction. Even though our classification is based on the current HAART regimen, we also considered the duration of exposure to the present treatment. The median duration of the current regimen was 18.5 months, which provides some insight into recent drug exposure. However, we acknowledge that classifying participants solely based on their current regimen does not account for prior treatment histories or cumulative exposure to different antiretroviral drug classes. Given the frequent switching of HAART regimens among PLWH, it is possible that some individuals categorized as non-PI or non-INSTI users may have previously received these drug classes. This limitation should be considered when interpreting the potential long-term impact of specific agents on sexual and gonadal function.

Although our study provides valuable insights, it is important to acknowledge its limitations. The main limitation is the relatively small sample size, as this may affect the statistical power and the generalizability of the findings. This constraint highlights the need for future studies with larger cohorts to confirm and extend our results. Additionally, being a single point evaluation, we are unable to capture changes over time and demonstrate if the observed relationships among variables remain constant. Moreover, another limitation could be the use of the IIEF-15 questionnaire, which was constructed and validated on heterosexual men. We did not employ the modified IIEF for men who have sex with men as only the English version is available [62]. Furthermore, we did not assess orgasmic function and intensity with dedicated tools [63, 64]. In addition, hormone levels were measured using chemiluminescence immunoassays, which, while commonly used in clinical practice, are less accurate than mass spectrometry, especially for low concentrations of analytes. The lack of more sensitive techniques, such as LC-MS/MS, represents a further limitation of the study. Moreover, while we assessed visceral fat accumulation using BMI and WC, we acknowledge that these measures may not accurately reflect body composition in PLWH. As this was a retrospective study, more accurate methods such as dual-energy X-ray absorptiometry (DEXA) or bioelectrical impedance analysis (BIA) were not available. This represents an additional limitation, and future prospective studies should include more specific tools for body composition analysis. The lack of a control group is another limit concerning the increased rate of prevalence of sexual dysfunction and hypogonadism in MLWH compared to the age-matched HIV-uninfected population. Therefore, further prospective studies are needed to better clarify certain aspects regarding the onset and progression of hypogonadism and sexual dysfunction in MLWH. However, our data highlights some interesting pathophysiological aspects underlying the fact that hypogonadism, which is also associated with increased visceral fat accumulation, is not the sole triggering factor for the onset of ED in MLWH.

## Conclusions

In conclusion, this study underscores the complex interplay among HIV infection, gonadal function, and sexual health in MLWH. Despite a significant prevalence of ED within the cohort, our findings suggest that the decrease in serum testosterone levels, often associated with increased visceral fat accumulation, is not the sole cause of its onset. HIV-related variables, particularly the type of HAART used, may also influence gonadal and sexual function. Overall, this study provides valuable insights into the multifaceted nature of gonadal and sexual health in MLWH, emphasizing the importance of tailored interventions to effectively address these issues. Consequently, the clinical management of sexual health in MLWH requires a multidisciplinary approach involving experts in infectious diseases and sexual medicine. Further investigations are needed to elucidate the mechanisms underlying sexual dysfunctions and gonadal impairment following the introduction of HAART.

## Supplementary Information

Below is the link to the electronic supplementary material.

Supplementary material 1 (DOCX 35.9 kb)

## Data Availability

The datasets generated during and/or analysed during the current study are available from the corresponding author on reasonable request.
