# Supplementary material for: Gonadal and sexual function in men living with HIV: insights from a single-centre study
Source: J Endocrinol Invest. 2025 Sep 22;48(12):2921–32. doi: 10.1007/s40618-025-02683-5 (PMC12640323; doi:10.1007/s40618-025-02683-5)
Supplement: Supplementary file 1 — Supplementary material 1 (DOCX 35.9 kb) [file 40618_2025_2683_MOESM1_ESM.docx]

**Table S1.** Clinical characteristics stratified for presence or absence of erectile dysfunction.

Continuous data are expressed as mean ± SD or median [IQR], as appropriate. Categorical variables are expressed as frequency (%). Boldfaced p values are significant.

Abbreviations: Human immunodeficiency virus (HIV); BMI, body mass index; WC, waist circumference; CD4^+^, CD4^+^ T helper cell count; TT, total testosterone; cFT, calculated free testosterone; SHBG, sex hormone binding globulin; LH, luteinizing hormone; E2, estradiol; ED, erectile disfunction; IIEF-15, International Index of Erectile Function-15; n.v., normal value.

|  | **Normal erectile function (n=18)** | **Erectile dysfunction (n=42)** | **p-value** |
| --- | --- | --- | --- |
| **Age (years)** | 56 ± 13 | 57 ± 8 | 0.854 |
| **Duration of HIV infection (years)** | 18 ± 10 | 20 ± 10 | 0.539 |
| **Active smoking (%)** | 6 (33.3) | 17 (40.4) | 0.759 |
| **BMI (kg/m²)** | 28.5 ± 5.9 | 28.4 ± 5.6 | 0.944 |
| **WC (cm)** | 104 ± 11 | 107 ± 18 | 0.121 |
| **CD4^+^ (cells/µL)** | 694 [516-808] | 662 [467-1152] | 0.778 |
| **TT (nmol/L)** | 18.3 ± 7.3 | 16.9 ± 7.1 | 0.525 |
| **cFT(nmol/L)** | 0.32 [0.19-0.43] | 0.33 [0.26-0.39] | 0.640 |
| **SHBG (nmol/L)** | 53.3 [33.9-63.2] | 45.8 [35.2-51.8] | 0.221 |
| **LH (mUI/mL)** | 5.2 [3.2-7.9] | 5.5 [3.7-7.1] | 0.926 |
| **E2 (pg/mL)** | 24.7 ± 8.5 | 26.4 ± 8.5 | 0.621 |
| **Hypogonadism (%)** | 3 (16.7) | 8 (19.0) | 0.560 |
| **IIEF-15 Erectile Function (n.v. >25)** | 26 [26-27] | 12 [5-20] | **<0.001** |
| **IIEF-15 Orgasmic Function (n.v. >9)** | 10 [10-11] | 6 [3-9] | **<0.001** |
| **IIEF-15 Sexual Desire (n.v. >9)** | 10 [10-11] | 6 [4-8] | **<0.001** |
| **IIEF-15 Intercourse Satisfaction (n.v. >9)** | 10 [10-11] | 3 [1-6] | **<0.001** |
| **IIEF-15 Overall Satisfaction (n.v. >9)** | 10 [10-11] | 6 [3-8] | **<0.001** |

**Table S2.** Clinical characteristics stratified for INSTI and non-INSTI users.

Continuous data are expressed as mean ± SD or median [IQR], as appropriate. Categorical variables are expressed as frequency (%). Boldfaced p values are significant.

Abbreviations: Human immunodeficiency virus (HIV); BMI, body mass index; WC, waist circumference; CD4^+^, CD4^+^ T helper cell count; TT, total testosterone; cFT, calculated free testosterone; SHBG, sex hormone binding globulin; LH, luteinizing hormone; E2, estradiol; ED, erectile disfunction; IIEF-15, International Index of Erectile Function-15; n.v., normal value.

|  | **INSTI users (n=43)** | **Non-INSTI users (n=17)** | **p-value** |
| --- | --- | --- | --- |
| **Age (years)** | 57 ± 9 | 54 ± 10 | 0.256 |
| **Duration of HIV infection (years)** | 19 ± 10 | 19 ± 9 | 0.866 |
| **Active smoking (%)** | 17 (39.5) | 6 (35.3) | 0.290 |
| **BMI (kg/m²)** | 28.5 ± 5.9 | 28.0 ± 5.1 | 0.825 |
| **WC (cm)** | 106.9 ± 18.3 | 104.8 ± 11.4 | 0.860 |
| **CD4^+^ (cells/µL)** | 638 [475-838] | 808 [549-1154] | 0.348 |
| **TT (nmol/L)** | 17.4 ± 7.5 | 16.9 ± 6.4 | 0.833 |
| **cFT(nmol/L)** | 0.33 [0.26-0.41] | 0.30 [0.24-0.35] | 0.487 |
| **SHBG (nmol/L)** | 47.0 [36.0-54.1] | 49.2 [29.5-57] | 0.630 |
| **LH (mUI/mL)** | 5.5 [3.97.2] | 3.5 [3.27.4] | 0.358 |
| **E2 (pg/mL)** | 25.7 ± 8.7 | 26.4 ± 7.0 | 0.869 |
| **Hypogonadism (%)** | 9 (20.9) | 2 (11.8) | 0.551 |
| **IIEF-15 Erectile Function (n.v. >25)** | 16 [10-26] | 26 [11-26] | 0.209 |
| **IIEF-15 Orgasmic Function (n.v. >9)** | 8 [4-10] | 10 [6-10] | 0.301 |
| **IIEF-15 Sexual Desire (n.v. >9)** | 7 [6-10] | 10 [6-10] | 0.185 |
| **IIEF-15 Intercourse Satisfaction (n.v. >9)** | 6 [2-10] | 10 [3-10] | 0.316 |
| **IIEF-15 Overall Satisfaction (n.v. >9)** | 8 [4-10] | 10 [6-10] | 0.301 |
| **ED (%)** | 35 (81.4) | 7 (41.2) | **0.017** |

**Table S3.** Clinical characteristics stratified for PI and non-PI users.

Continuous data are expressed as mean ± SD or median [IQR], as appropriate. Categorical variables are expressed as frequency (%). Boldfaced p values are significant.

Abbreviations: Human immunodeficiency virus (HIV); BMI, body mass index; WC, waist circumference; CD4^+^, CD4^+^ T helper cell count; TT, total testosterone; cFT, calculated free testosterone; SHBG, sex hormone binding globulin; LH, luteinizing hormone; E2, estradiol; ED, erectile disfunction; IIEF-15, International Index of Erectile Function-15; n.v., normal value.

|  | **PI users (n=7)** | **Non-PI users (n=53)** | **p-value** |
| --- | --- | --- | --- |
| **Age (years)** | 55 ± 5 | 57 ± 10 | 0.736 |
| **Duration of HIV infection (years)** | 23 ± 10 | 19 ± 9 | 0.168 |
| **Active smoking (%)** | 5 (71.4) | 18 (34.0) | 0.074 |
| **BMI (kg/m²)** | 23.5 ± 2.3 | 28.7 ± 5.6 | 0.208 |
| **WC (cm)** | 106.2 ± 12.3 | 104.1 ± 11.4 | 0.760 |
| **CD4+ (cells/µL)** | 686 [617-920] | 666 [467-917] | 0.701 |
| **TT (nmol/L)** | 23.7 ± 7.4 | 16.5 ± 6.8 | **0.018** |
| **cFT(nmol/L)** | 0.45 [0.28-0.83] | 0.32 [0.24-0.39] | 0.268 |
| **SHBG (nmol/L)** | 59.6 [55.6-95.5] | 45.1 [29.5-52.5] | **0.015** |
| **LH (mUI/mL)** | 8.4 [3.5-14.0] | 5.2 [3.4-7.1] | 0.332 |
| **E2 (pg/mL)** | 39.8 ± 2.0 | 24.5 ± 7.5 | **0.020** |
| **Hypogonadism (%)** | 0 (0) | 11 (20.7) | 0.189 |
| **IIEF-15 Erectile Function (n.v. >25)** | 25 [5-27] | 18 [11-26] | 0.425 |
| **IIEF-15 Orgasmic Function (n.v. >9)** | 10 [4-11] | 9 [6-10] | 0.391 |
| **IIEF-15 Sexual Desire (n.v. >9)** | 10 [4-11] | 8 [6-10] | 0.391 |
| **IIEF-15 Intercourse Satisfaction (n.v. >9)** | 9 [1-11] | 6 [3-10] | 0.505 |
| **IIEF-15 Overall Satisfaction (n.v. >9)** | 9 [2-11] | 9 [4-10] | 0.776 |
| **ED (%)** | 4 (57.1) | 38 (71.7) | 0.207 |
| **Impaired orgasmic function (%)** | 3 (42.9) | 36 (67.9) | 0.233 |
| **Impaired sexual desire (%)** | 3 (42.9) | 38 (71.7) | 0.197 |
| **Impaired intercourse satisfaction (%)** | 3 (42.9) | 40 (75.5) | 0.176 |
| **Impaired overall satisfaction (%)** | 4 (57.1) | 37 (68.8) | 0.670 |
